# Visual Learning in Multiple-Object Tracking

**DOI:** 10.1371/journal.pone.0002228

**Published:** 2008-05-21

**Authors:** Tal Makovski, Gustavo A. Vázquez, Yuhong V. Jiang

**Affiliations:** 1 Department of Psychology, University of Minnesota, Minneapolis, Minnesota, United States of America; 2 Department of Psychology, University of Santiago, Santiago, Spain; 3 Center for Cognitive Sciences, University of Minnesota, Minnesota, United States of America; Ecole Polytechnique Federale de Lausanne, Switzerland

## Abstract

**Background:**

Tracking moving objects in space is important for the maintenance of spatiotemporal continuity in everyday visual tasks. In the laboratory, this ability is tested using the Multiple Object Tracking (MOT) task, where participants track a subset of moving objects with attention over an extended period of time. The ability to track multiple objects with attention is severely limited. Recent research has shown that this ability may improve with extensive practice (e.g., from action videogame playing). However, whether tracking also improves in a short training session with repeated trajectories has rarely been investigated. In this study we examine the role of visual learning in multiple-object tracking and characterize how varieties of attention interact with visual learning.

**Methodology/Principal Findings:**

Participants first conducted attentive tracking on trials with repeated motion trajectories for a short session. In a transfer phase we used the same motion trajectories but changed the role of tracking targets and nontargets. We found that compared with novel trials, tracking was enhanced only when the target subset was the same as that used during training. Learning did not transfer when the previously trained targets and nontargets switched roles or mixed up. However, learning was not specific to the trained temporal order as it transferred to trials where the motion was played backwards.

**Conclusions/Significance:**

These findings suggest that a demanding task of tracking multiple objects can benefit from learning of repeated motion trajectories. Such learning potentially facilitates tracking in natural vision, although learning is largely confined to the trajectories of attended objects. Furthermore, we showed that learning in attentive tracking relies on relational coding of all target trajectories. Surprisingly, learning was not specific to the trained temporal context, probably because observers have learned motion *paths* of each trajectory independently of the exact temporal order.

## Introduction

Tracking moving objects in space is important for the maintenance of spatiotemporal continuity in everyday visual tasks, such as sports, driving, and keeping track of children on the playground. When a salient feature such as color distinguishes the targets from nontargets, tracking is easily achieved by remembering the target feature. However, when salient featural differences are absent, tracking must rely on attention. The latter case is exemplified by the multiple-object tracking (MOT) task, where several visually identical objects move randomly on a display and the observers track with attention a prespecified subset of objects [1]–[2]. Research using this task reveals that humans can track about four objects among other objects moving at moderate speeds, but performance declines with increasing target number, increasing motion speed, and decreasing object-to-object distance [3]–[4].

Although attentive tracking appears quite limited in laboratory settings, the limitation may be alleviated in daily activities. Objects in natural vision do not move in a completely unpredictable manner. Repeated exposure to a given visual environment, such as the same driving route with fixed lanes, may enhance tracking. Indeed, recent research has shown that humans are highly sensitive to repetitions in the visual input. They are faster at finding a target on search displays that repeat occasionally. Such learning, known as “contextual cueing,” is observed when searching for a static target [5] or a moving target [6] among repeated search displays. Visual associative learning is also seen for shapes that frequently co-occur in space or in temporal sequence [7]–[8].

The prevalence of statistical learning in visual tasks suggests that attentive tracking may be similarly influenced by learning. However, learning in a MOT task is more challenging than in other tasks. In MOT, objects constantly change locations, providing limited opportunities for observers to learn from any instance of motion. Furthermore, the task places strong demands on the observer's ability to simultaneously learn several target trajectories. This demand may not be easily met. For example, contextual cueing is rapidly acquired when a search display is consistently associated with one target location, but it fails to develop if a search display is associated with four target locations [9]. Nonetheless, a recent technical report demonstrated some evidence for learning in MOT [10].

This study aims to systematically characterizing visual learning in attentive tracking. We are interested in MOT learning because the MOT task uniquely taps into various aspects of attention in a single paradigm. In this task, attention is *divided* among multiple targets, which must be *selected* from nontargets and *maintained* across *spatial* and *temporal* changes. These properties make the MOT task a perfect candidate for characterizing how varieties of attention interact with visual learning. The goal of this paper is to address the following questions.

First, how does selective attention constrain visual learning in MOT? Previous studies that investigate the role of attention in learning have often used tasks that exert minimal requirement on selective attention. For example, the serial reaction time task [11] presents observers with a sequence of trials, where each trial involves only one stimulus, eliminating any need to select the target from distractors. In contrast, the MOT task is inherently a selective attention task. It is well suited to address whether learning is constrained by selective attention. To this end, we investigate whether attended and unattended trajectories are learned equally well.

Secondly we ask whether target trajectories are learned in relation to one another or as separate, independent motion trajectories. This issue is important as it can shed light on a recent debate in the literature. Namely, when attention is divided among multiple target trajectories, are the different attentional foci fully independent or are they inter-related? This question has proven difficult to answer, with some researchers proposing independent “pointers” for tracking separate targets [12], while others proposing a single spotlight or multiple interdependent spotlights for all targets [13]. This study addresses this question from the perspective of visual learning.

Finally, because attention is deployed not only in space but also in time, the MOT task allows us to test the specificity of learning to trained temporal context. In other tasks, such as visual search through a sequence of centrally-presented letters, participants usually learn the temporal order of stimuli and use it to predict what comes next [14]. The MOT task also involves temporally sequenced stimuli, yet it has an additional spatial component. It is therefore of interest to test whether learning in a spatiotemporal task reveals the same kind of temporal specificity as learning in a purely temporal task. To this end, we examine whether learning in MOT transfers to presentation of learned motion sequence presented backwards.

### The current study

Participants tracked four moving circles within a field of eight moving circles. The motion trajectories were repeatedly presented during training. To prevent participants from learning just the final positions of a motion trial, the trials were terminated at a randomly selected moment. We then tested participants in a transfer session where trials with novel trajectories (*new*) were compared with trials with previously experienced trajectories. In the transfer session of Experiment 1 (Table 1), sometimes the tracked subset was the same as that used during training (*old*), sometimes it was the opposite from that used during training (*target-distractor switched*), sometimes it included two of previously tracked targets and two of previously ignored distractors (*mixed*). This design allowed us to address several theoretical questions.

**Table 1:** An illustration of the conditions tested in Experiment 1.

Condition	Target set	Distractor set
Training (15 times)	[1], [2], [3], [4]	[5], [6], [7], [8]
Transfer - Old	[1], [2], [3], [4]	[5], [6], [7], [8]
Transfer - Switched	[5], [6], [7], [8]	[1], [2], [3], [4]
Transfer - Mixed	[1], [2], [5], [6]	[3], [4], [7], [8]
Transfer - New	[9], [10], [11], [12]	[13], [14], [15], [16]

The numbers 1 to 16 correspond to 16 random motion trajectories.

First, if learning is constrained by selective attention, such that only attended trajectories are learned, then learning should transfer only when the target trajectories in the transfer phase matched those used during training. Consequently, performance in the *old* condition should be high but that in the *switched* condition should be low. Alternatively, if learning is independent of selective attention, then all trajectories should be learned equally well. Consequently, learning should transfer equally well to the *switched* and the *old* conditions.

Second, with regard to what is being learned, the comparison between the *mixed* condition and other conditions can inform us whether multiple target trajectories are learned independently of one another. If so, repeating two of the target trajectories should lead to about half as much transfer as repeating all four target-trajectories. Alternatively, if the target trajectories are learned in relation to one another, then transfer to the *mixed* condition should be largely eliminated. Here we did not consider the possibility of negative transfer from distractor trajectories; this will be discussed later.

Experiment 2 further addresses the specificity of learning by asking whether learning is specific to the trained *temporal* context. Many forms of visual statistical learning involve repeated temporal order. Such learning allows participants to predict the target object based on the preceding objects [14]. The motion trajectories used in MOT are temporally ordered, making it possible that learning is specific to the trained temporal context. However, in this task temporal information is also integrated with spatial information. Thus, the trajectories form motion *path*, a spatially arrayed trajectory. It is possible that MOT learning is partly comprised of learning of the motion *path* independently of the exact temporal order (or “vector” of motion). Experiment 2 tests these competing possibilities by examining whether learning transfers to learned motion played backward in time.

## Materials and Methods

### Participants

The study was approved by the University of Minnesota IRB Human Subjects Committee. Participants were volunteers from the University of Minnesota. They were 18 to 35 years old, had normal color vision, and normal or corrected-to-normal visual acuity. All participants provided informed consent and received one course credit or $10/hr. Twelve participants (mean age 20.7 years) completed Experiment 1 and fifteen new participants (mean age 20.9 years) took part in Experiment 2.

### Stimuli

The moving objects were circles 0.6° in diameter presented against a gray background. All had the same color and size on a given trial, but the exact color could be one of eight salient colors and was randomly determined on each trial.

### Procedure

Participants initiated each trial by pressing the spacebar, which brought up 8 objects presented at randomly selected locations within an imaginary square (21°×21°). The objects were stationary during the cue period with four cued by an outline white square (1.0°×1.0°). The cue lasted for 1330 msec, after which the white squares disappeared and the objects moved at a constant speed. Participants were asked to track the cued objects and were encouraged to maintain fixation during tracking. The objects bounced off the edge of the imaginary square or repelled one another at a minimal center-to-center distance of 1.2°. After 6–8 s of motion, the objects stopped and turned white. Participants responded by clicking on four items, after which the correctly clicked targets turned green and the missed targets turned red for 0.5 s.

### Experiment 1's Design

The experiment contained three consecutive phases. During *training*, participants completed 15 blocks, each including 8 different trials. Each trial was 8 s in motion duration and was shown in its entirety in Block 1. Subsequently, these eight trials were repeated once per block for the remaining blocks with random trial orders. Each trial terminated at a randomly determined time after 6–7.5 s of motion in Blocks 2 to 15. Because objects moved at 22.5°/sec, each object would have moved up to 33.8° during a 1.5 s window. The ending configuration was highly dissimilar from one repetition to another, making it impossible for participants to learn just the configuration of the ending display.

The *transfer* phase commenced immediately after training without special instructions. Trials used in the transfer phase were all novel trajectories (*new* condition), or the same trajectories as used during training. There were three ways in which the eight objects of the old trials might be cued. In the *old* condition, the same subset of objects previously cued as targets during training, were cued. In the *switched* condition, the previously uncued objects were cued as tracking targets. In the *mixed* condition, two previously cued objects and two previously uncued objects were cued as tracking targets. The 8 trials in each of the four conditions were presented in a random order. To increase statistical power, the same 32 trials (including the same new trials) were presented again (in a different order). Similar to training, a transfer trial terminated at a random moment after 6–7.5 s of motion.

Following the transfer phase, participants were tested in a *recognition* phase, where they were shown the 32 transfer trials along with 32 matching trials whose starting displays (i.e., the cue period) were the same as the 32 transfer trials, but whose motion trajectories were newly generated. On each trial during *recognition*, participants tracked the cued targets and reported whether the trial contained trajectories they saw before.

### Experiment 2's design

There were 20 training blocks in Experiment 2. During block 1, 6 unique trials (each with 8 objects) were randomly generated for each participant. Objects moved at a constant speed of 17.5°/sec for 8.5 s in Block 1. Blocks 2 to 20 contained the same 6 trials presented in a random order, but the trials started at a random point of time between 0–2.5 s of Block 1's starting time and lasted 6 s. The randomization of the starting positions ensured that across repetitions, trials would start and end with highly divergent displays.

Participants were tested in 3 conditions in the *transfer* phase. The *new* condition used new motion trajectories for all objects. The *old*, *forward-motion* condition was the same as training, while the *old*, *backward-motion* condition was the trained trials played backwards. The duration of motion was held constant at 6 seconds in all conditions. Each trial started at a random moment 0–2.5 s from the beginning (or 0–2.5 s from the end, when motion was played backward) of a trial. The designation of targets in the *old* conditions always matched that of training. There were 6 trials in each condition to form 18 trials of a transfer block. To increase statistical power, we repeated these 18 trials for a second transfer block.

Finally, the same 18 trials used in the transfer block were presented again in the *recognition* phase. Participants were asked to track cued targets and report whether the trial contained repeated trajectories. All other aspects of the experiment were the same as those of Experiment 1.

## Results

Accuracy in a given trial was calculated by averaging the responses given to all four targets. For example, if a participant correctly clicked on 3 out of the 4 targets, accuracy would be 75% on that trial.

### Experiment 1

#### 1. Training

**Figure 1:**
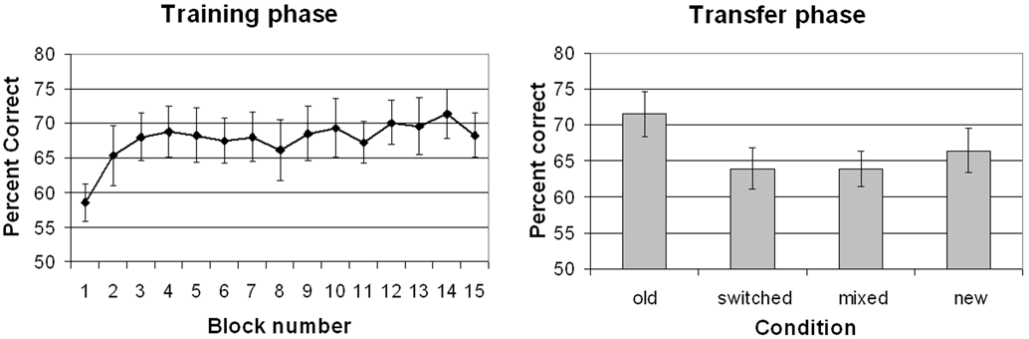
Experiment 1 Results. Left: Training phase. Right: Transfer phase. Error bars show ±1s.e.

Tracking in Block 1 was marginally poorer than in Block 2 (Figure 1, left), *F*(1, 11) = 4.31, *p* = .06. This difference might reflect very rapid learning, or more plausibly, effects of different tracking duration (8 s in Block 1 versus an average of 6.75 s in later blocks). Trial duration was comparable in blocks 2 to 15, and accuracy increased numerically but not statistically. The linear improvement from Blocks 2 to 15 failed to reach significance, *F*(1, 11) = 2.18, *p*>.17. The lack of significant improvement during training does not indicate an absence of learning, as training effects may be masked by fatigue or reduced motivation at later points of the training session. It is therefore important to assess learning by comparing performance between old, new, and other conditions in a single transfer phase.

#### 2. Transfer

Accuracy in the transfer phase (Figure 1, right) revealed a significant main effect of condition, *F*(3, 33) = 6.85, *p*<.01. The *old* condition was more accurate than the *new* condition, *F*(1, 11) = 5.08, *p*<.05. The *switched* and the *mixed* conditions were both significantly worse than the *old*, *F*(1, 11)s>18.61, *ps*<.01, and not significantly different from each other or from the *new* condition, *F*(1, 11)s<1.98, *p*s>.15. This pattern was observed in each of the two transfer blocks, as block did not interact with transfer condition, *F*<1.

#### 3. Recognition

When queried after the transfer phase, half of the participants reported that they had noticed the repetition of motion trajectories. However, these “aware” participants were no more likely than the others at judging whether an old trial (or an entirely novel trial) was previously repeated or not, *F*<1. “Aware” participants also did not show greater benefit from the *old* condition during the transfer phase, *F*<1. The following analyses were pooled across all participants.

**Figure 2:**
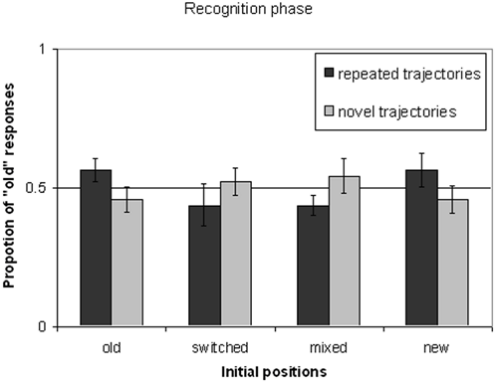
Recognition data from Experiment 1.

Forced choice of whether a trial involved repeated or novel trajectories revealed no strong evidence for the presence of explicit knowledge (Figure 2). Trials with repeated trajectories were no more likely than trials with novel trajectories to receive a response of “old,” *F*<1, when the two conditions share the same cueing phase. The two conditions most extreme in terms of novelty were not different in recognition response. The percentage of “old” responses was comparable for repeated displays with *old* initial displays and for new trajectories with novel initial displays, *t*(11) = 1.60, *p*>.14.

### Experiment 2

#### 1. Training

**Figure 3:**
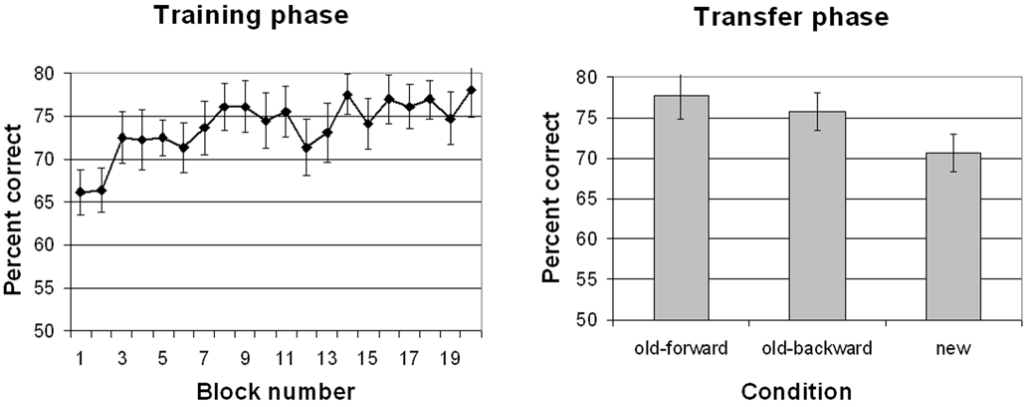
Results from Experiment 2. Left: training phase. Right: transfer phase. Error bars show ±1s.e.

Figure 3 (left panel) shows that accuracy improved as training progressed from blocks 2 to 20 (we excluded Block 1 because its motion duration was longer), resulting in a significant linear trend of block, *F* (1, 14) = 14.52, *p*<.01. The stronger learning seen in this experiment compared with Experiment 1 may be attributed to the use of fewer unique trials and more repetitions.

#### 2. Transfer

Performance in the *old*, *forward-motion* condition was significant higher than that in the *new* condition, *F*(1, 14) = 13.46, *p*<.01. This learning transferred to the *old*, *backward-motion* condition, which was significantly more accurate than the *new* condition, *F*(1, 14) = 5.66, *p*<.05, but not significantly different from the *old*, *forward-motion* condition, *F*(1, 14) = 1.59, *p*>.22. This pattern was seen in both transfer blocks, as the interaction between transfer block and condition was insignificant, *F*<1.

#### 3. Recognition

Participants were significantly more likely to report a display as containing repeated trajectories in the *old*, *forward-motion* condition (M = 56.7%) than the *new* condition (M = 35.6%), *F* (1, 14) = 8.23, *p*<.05. The *old*, *backward-motion* condition was intermediate (M = 47.8%), not significantly different from the *forward-motion*, *F*(1, 14) = 1.20, *p*>.29, or the *new* condition *F*(1, 14) = 3.90, *p* = .07. Individual subjects' results showed, however, that participants who recognized more repeated displays did not show greater MOT learning in the transfer phase, *F*<1. Thus, while there was evidence for explicit recognition of repeated displays, it was unclear whether improvement in the tracking task was driven exclusively by explicit knowledge.

## Discussion

This study shows that the human visual system is capable of learning in an attentive tracking task, even though the visual stimuli are complex and the task is attentionally demanding. Experiment 1 demonstrated that visual learning could assist attentive tracking, even though there were multiple target trajectories to learn [10]. Learning did not transfer to trials with the same motion trajectories but different designation of targets and nontargets, suggesting that learning in attentive tracking does not originate from increased familiarity with repeated motion trajectories. The lack of transfer in the target-distractor *switched* condition is particularly notable. It suggests that learning was not driven by an improved ability to divide the moving objects into two specific subsets. That is, attentional segregation in MOT is not a simple matter of dividing the display into two subsets; it is more analogous to figure-ground segregation where the reversal of figure and ground produces a new percept. These results indicate that MOT learning is constrained by selective attention.

With regard to what is learned, Experiment 1 also shows that the multiple target trajectories are learned in relation to one another. This is because there was no partial transfer to the *mixed* condition where two former targets and two former distractors were to be tracked. But could the lack of partial transfer be explained by a cancellation between positive transfer to the targets and negative transfer to the nontargets? That is, suppose that suppression of the distractor trajectories led to negative transfer, which may have cancelled out partial transfer from two of the target trajectories. The net result would be a lack of improvement in the *mixed* condition. The notion of negative transfer is appealing but is unsubstantiated by our data. Any negative transfer should be revealed most strongly in the *switched* condition, yet our data showed that performance in the *switched* condition was not worse than the *mixed* condition, providing no evidence for the cancellation of negative and positive transfers in the latter. Instead, the lack of partial transfer in the *mixed* condition is most consistent with the idea that multiple attentional trajectories are processed in relation to one another [13].

Although MOT learning is specific to the learned spatial context, it is not highly specific to the trained temporal context. Experiment 2 showed that learning readily (though perhaps not fully) transfers to trials with motion played backwards, suggesting that the vector of motion was not a critical component of learning. What enabled learning to transfer to backwards motion? There are at least two possibilities: participants might have learned the snapshots of the spatial configuration for each moment of motion, or they might have learned the motion path of each trajectory. Both the moment-to-moment snapshots and the motion path were the same for forward and backward motion. Although our experiment could not distinguish between these two possibilities, we believe that the snapshot account is highly implausible. The snapshot account requires participants to learn four target locations for each snapshot, yet a previous study has shown that participants are unable to associate a search display with four target locations [9]. These findings also support a recent claim that backward predictions and forward predictions are comparable in associative learning [15]. They suggest that temporal prediction (such as extrapolation of future motion locations) is a not critical component of attentive tracking [16].

Is the kind of learning involved in MOT the same as that involved in other type of visual statistical learning? A direct answer to this question would require tests of transfer across tasks, such as transfer from trained motion trajectories to visual search of moving objects in those trajectories. Without such data, we can only make speculative comparisons. Learning in MOT is similar to other types of visual learning in its sensitivity to selective attention. However, it is difficult to conceive MOT learning simply as snapshots of spatial context learning. The number of configurations involved in each trial is much greater in tracking, and the number of targets also exceeds that usually learned in visual search [9]. Furthermore, learning in MOT relies on relational coding of all target trajectories, whereas learning of repeated spatial context depends on individual locations [17]. Whether the same type of visual learning is used for attentive tracking as for other tasks remains to be seen.

Our conclusion that learning in attentive tracking is gated by attention is consistent with Ogawa and Yagi's [10] finding that repeating distractors alone was not beneficial. However, Ogawa and Yagi also found that tracking was more accurate when both targets and distractors repeated, than when only the targets repeated. This finding is not inconsistent with the attention-dependent account, as it needs not imply that distractors are learned. Instead, the utility of target-repetition may be lower when distractor trajectories are novel. With novel distractor trajectories, there are new uncertainties as when distractors may get closer to the targets, and those are the moments when observers are most likely to lose the targets. The interaction between targets and distractors is thus more variable when distractors are new, reducing the utility of target learning.

In summary, this study has shown that a demanding task of tracking multiple objects can benefit from learning of repeated motion trajectories. Such learning potentially facilitates tracking in natural vision, although learning is largely confined to the trajectories of attended objects.
